# Fiscal policy to improve diets and prevent noncommunicable diseases: from recommendations to action

**DOI:** 10.2471/BLT.17.195982

**Published:** 2018-02-05

**Authors:** Anne Marie Thow, Shauna M Downs, Christopher Mayes, Helen Trevena, Temo Waqanivalu, John Cawley

**Affiliations:** a Menzies Centre for Health Policy, School of Public Health, Charles Perkins Centre (D17), University of Sydney, New South Wales, 2006, Australia.; b Department of Health Systems and Policy, Rutgers School of Public Health, New Brunswick, United States of America (USA).; c Alfred Deakin Institute for Citizenship and Globalisation, Deakin University, Geelong, Australia.; d Prevention of Noncommunicable Diseases Department, World Health Organization, Geneva, Switzerland.; e Department of Policy Analysis and Management, Cornell University, New York, USA.

## Abstract

The World Health Organization has recommended that Member States consider taxing energy-dense beverages and foods and/or subsidizing nutrient-rich foods to improve diets and prevent noncommunicable diseases. Numerous countries have either implemented taxes on energy-dense beverages and foods or are considering the implementation of such taxes. However, several major challenges to the implementation of fiscal policies to improve diets and prevent noncommunicable diseases remain. Some of these challenges relate to the cross-sectoral nature of the relevant interventions. For example, as health and economic policy-makers have different administrative concerns, performance indicators and priorities, they often consider different forms of evidence in their decision-making. In this paper, we describe the evidence base for diet-related interventions based on fiscal policies and consider the key questions that need to be asked by both health and economic policy-makers. From the health sector’s perspective, there is most evidence for the impact of taxes and subsidies on diets, with less evidence on their impacts on body weight or health. We highlight the importance of scope, the role of industry, the use of revenue and regressive taxes in informing policy decisions.

## Résumé

L'Organisation mondiale de la Santé a recommandé aux États membres d'envisager de taxer les boissons et aliments à haute teneur énergétique et/ou de subventionner les denrées riches en nutriments, en vue d'améliorer les régimes alimentaires et de prévenir les maladies non transmissibles. Aujourd'hui, nombreux sont les pays à avoir instauré des taxes sur les boissons et aliments à haute teneur énergétique ou à envisager de le faire. Néanmoins, d'importants défis subsistent pour la mise en application de ce type de politiques fiscales. Certains sont liés à la nature intersectorielle des interventions appropriées. Par exemple, comme les responsables des politiques économiques et les responsables des politiques de santé ont des préoccupations administratives, des priorités et des indicateurs de performances différents, ils s'appuient souvent sur différentes formes de données dans leur prise de décisions. Dans le présent document, nous décrivons les données probantes susceptibles d'orienter les interventions sur l'alimentation fondées sur des politiques fiscales et nous évoquons les principales problématiques auxquelles doivent répondre à la fois les responsables des politiques économiques et les responsables des politiques de santé. D'un point de vue de santé publique, les preuves de l'impact des taxes et subventions sur les habitudes alimentaires sont plus nombreuses que les preuves de leur impact sur le poids ou la santé. Nous abordons également l'importance du périmètre d'action, le rôle de l'industrie, l'utilisation des recettes fiscales et la régressivité des taxes, dans l'optique d'éclairer les décisions politiques.

## Resumen

La Organización Mundial de la Salud ha recomendado a los Estados Miembros considerar la posibilidad de aplicar un impuesto a las bebidas y los alimentos de alto contenido energético y/o subvencionar los alimentos ricos en nutrientes para mejorar las dietas y prevenir enfermedades no contagiosas. Numerosos países ya aplican impuestos a bebidas y alimentos de alto contenido energético o consideran la implementación de dichos impuestos. Sin embargo, persisten varios desafíos importantes para la implementación de políticas fiscales para mejorar las dietas y prevenir las enfermedades no contagiosas. Algunos de estos desafíos están relacionados con la naturaleza intersectorial de las intervenciones correspondientes. Por ejemplo, puesto que los encargados de la formulación de políticas de salud y economía tienen diferentes preocupaciones administrativas, indicadores de rendimiento y prioridades, a menudo tienen en cuenta diferentes formas de pruebas en su toma de decisiones. En este documento, se describe la base de pruebas para intervenciones relacionadas con la dieta basadas en políticas fiscales y se consideran las preguntas clave que deben formular tanto los responsables de la política económica como de la de salud. Desde la perspectiva del sector de la salud, existen muchas pruebas del impacto de los impuestos y subsidios en las dietas, con menos pruebas de sus impactos sobre el peso o la salud corporal. Se destaca la importancia del alcance, el papel de la industria, el uso de los ingresos y los impuestos regresivos para informar sobre las decisiones políticas.

## ملخص

أوصت منظمة الصحة العالمية الدول الأعضاء بدراسة فرض ضرائب على المشروبات والأطعمة كثيرة السعرات الحرارية و/أو دعم الأطعمة الغنية بالمغذيات وذلك من أجل تحسين الأنظمة الغذائية ومقاومة الأمراض غير المعدية. وقد قامت العديد من البلدان بفرض الضرائب بالفعل على المشروبات والأطعمة كثيرة السعرات الحرارية أو تدرس فرض ضرائب من هذا النوع، إلا أن العديد من التحديات الرئيسية تواجه تنفيذ السياسات المالية لتحسين الأنظمة الغذائية والوقاية من الأمراض غير المعدية. وتتعلق بعض هذه التحديات بطبيعة التدخلات ذات الصلة والمشتملة على عدة قطاعات. على سبيل المثال، فإن تباين الاهتمامات الإدارية ومؤشرات الأداء والأولويات لدى مقرري السياسات الاقتصادية والصحية غالبًا ما يدفعهم إلى اعتماد أشكال مختلفة للأسس الدلالية التي يستندون إليها في عملية اتخاذ القرار. ونصف في هذا التقرير الأساس الدلالي للتدخلات ذات الصلة بالأنظمة الغذائية والمعتمدة على السياسات المالية، ونبحث الأسئلة الرئيسية الوارد طرحها من جانب من كلٍ من مقرري السياسات الاقتصادية والصحية. ومن منظور القطاع الصحي تشير معظم الدلائل إلى حدوث تأثير ناتج عن فرض الضرائب والدعم على الأنظمة الغذائية، في حين توجد دلائل أقل تشير إلى تأثيرها على وزن الجسم أو الصحة. ونحن نقوم بتسليط الضوء على أهمية النطاق ودور الصناعة والاستفادة من الإيرادات والضرائب التنازلية في اتخاذ القرارات المستنيرة المتعلقة بالسياسات.

## 摘要

世界卫生组织建议会员国考虑对高能量饮料和食品征税和/或给予富含营养物质的食品补贴，以改善饮食和预防非传染性疾病。许多国家要么已经对高能量饮料和食品征税，要么正在考虑实施此项税赋。但是，实施改善饮食和预防非传染性疾病的财政政策仍然面临若干重大挑战。其中一些挑战与相关干预的跨部门性质有关。例如，由于卫生和经济决策者的行政关注、绩效指标和优先事项有所不同，决策者在制定决策时往往会考虑不同形式的依据。在本文中，我们描述了基于财政政策的饮食相关干预的依据基础，并考虑了卫生和经济决策者都需要提出的关键问题。从卫生部门的角度来看，很多依据可以证明税收和补贴对饮食的影响，但很少有依据可以证明其对体重或健康的影响。我们强调了范围、行业角色、营业税和累退税的使用在传达政治决策中的重要性。

## Резюме

Всемирная организация здравоохранения рекомендовала государствам-членам рассмотреть вопрос об обложении налогом высококалорийных напитков и продуктов питания и (или) субсидировании богатых питательными веществами продуктов питания для улучшения рациона и профилактики неинфекционных заболеваний. Многие страны либо применяют налоги на высококалорийные напитки и продукты питания, либо рассматривают возможность применения таких налогов. Тем не менее остается несколько серьезных проблем, связанных с осуществлением налогово-бюджетной политики, направленной на улучшение рациона и профилактику неинфекционных заболеваний. Некоторые из этих проблем связаны с межсекторальным характером соответствующих интервенций. Например, поскольку лица, определяющие политику в области здравоохранения и экономики, имеют разные по характеру административные проблемы, показатели эффективности и приоритеты, они часто учитывают различные формы доказательных данных в процессе принятия решений. В этой статье мы описываем доказательную базу для связанных с рационом интервенций на основе налогово-бюджетной политики и рассматриваем ключевые вопросы, которыми должны задаваться лица, определяющие политику в области здравоохранения и экономики. С точки зрения сектора здравоохранения имеется больше данных о влиянии налогов и субсидий на рацион при меньшем объеме данных об их воздействии на вес тела или здоровье. Мы подчеркиваем важность сферы применения, роли промышленности, использования доходов и регрессивных налогов для принятия политических решений.

## Introduction

In 2016, the World Health Organization (WHO) recommended the “implementation of an effective tax on sugar-sweetened beverages” as one of several key measures to address childhood obesity.^1^ This reflected the conclusions of a WHO Technical Meeting in 2015 that focused on fiscal policies for improving diets and preventing noncommunicable diseases.^2^

The economic and social costs of noncommunicable diseases include not just the direct costs of the relevant health care, but also many indirect costs, e.g. those associated with higher job absenteeism.^3^ There are strong economic and health rationales for using fiscal policies to improve diets and prevent such diseases. Fiscal interventions can play a key role in correcting for market failure, particularly when the price of a product does not fully reflect its full social cost. Fiscal policies can be used to alter retail prices in such a way that sales and consumption of foods associated with the development of noncommunicable diseases are reduced.^4^

As the evidence of the potential benefits of such policies to public health has increased, the development of fiscal policies to improve diets and prevent noncommunicable diseases has begun to gain momentum.^2^^,^^5^ By December 2017, the NOURISHING framework had recorded the introduction of such policies in at least 29 jurisdictions: 14 national policies had been introduced in low- or middle-income countries and 15 national or more local policies had been introduced in high-income countries (Box 1).^6^ In some countries, however, the implementation of fiscal interventions to improve diets has faced challenges. In Denmark, for example, a tax on saturated fat was removed after one year.^7^ In one county in the United States of America, a tax on sugar-sweetened beverages was repealed after less than a year.^8^ In South Africa, the sugar industry actively lobbied against the introduction of a tax on soft drinks^9^ and, in Fiji, a tax on soft drinks was reduced and revised after the soft-drinks industry complained about the irregularity of the taxation’s enforcement.^10^

Box 1 **Jurisdictions with health-related fiscal policies applied to foods and/or beverages, 2017**

**Taxes on sugar-sweetened beverages**

Implemented nationally in Barbados, Belgium, Brunei Darussalam, Chile, Fiji, Finland, France, Kiribati, Mauritius, Mexico, Norway, Samoa, Saudi Arabia, Spain, Tonga, Vanuatu and locally in British Overseas Territory Saint Helena and within the United States of America, in Albany, Berkeley, Boulder, Oakland, Philadelphia and the Navajo Nation.

**Taxes on foods high in salt, fat and/or sugar**

Implemented nationally by Dominica, Hungary, Saint Vincent and the Grenadines and Tonga and locally, in French Polynesia and by the Navajo Nation in the United States.

**Subsidies to improve diets and health**

Targeted subsidies have been embedded into social welfare programmes within the United Kingdom of Great Britain and Northern Ireland and the United States, targeted to remote populations in Canada and provided by private health insurance programmes in South Africa. Implicit subsidies have been granted, through removal of import tariffs on fruit and vegetables, in Fiji and Tonga.

Source: World Cancer Research Fund International’s NOURISHING framework.^6^

The development of an effective fiscal policy to improve diets has to take account of political economy as well as the potential benefits to public health. Therefore, health and finance policy-makers need to collaborate in their design. Policy-makers in the health sector are mainly interested in the effectiveness of policies for improving population health through changes to diets. Policy-makers in the finance sector have a somewhat different focus that is related to their own agendas and administrative responsibilities.^11^ They may want to know whether an excise tax would be better than a sales tax, how the tax could be administered most effectively and the likely impact of the tax on government revenue, employment, industry and livelihoods.

Both groups of policy-makers may also be concerned about regressivity, i.e. whether the tax will disproportionately fall on lower-income individuals. Both may also be concerned about how revenue from the tax is earmarked and spent; health policy-makers may prefer such revenue to be spent on further promoting health, whereas policy-makers in the finance sector may prefer to treat it as general revenue that can be spent without constraint.

In this paper, we analyse key health and economic policy considerations arising from recent recommendations on, and the implementation of, fiscal policies to improve diets. Our analysis is also informed by the multidisciplinary literature relevant to interventions, based on fiscal policies, as well as research on implementation in practice, and highlights gaps in the evidence base that need to be filled by future research.

## Evidence-based policy design

### Identification of targets

Policy-makers in the health sector should consider three key aspects when identifying appropriate targets for taxes or subsidies. First, according to the available epidemiological evidence, which foods and nutrients are associated with poorer, or better, health outcomes. Second, the extent to which consumption of the relevant foods or nutrients is likely to impose negative externalities on society, and the extent to which consumption is likely to be affected by taxes and subsidies. Third, which targets are likely to be the most feasible, from an administrative perspective.

#### Improving health

Strong evidence indicating that the risk of developing diet-related noncommunicable diseases could be reduced by decreasing the consumption of added sugars, red and processed meats, refined grains, salt, sugar-sweetened beverages and/or trans-fat and/or by increasing the consumption of fish, fruits, legumes, minimally processed whole grains, non-starchy vegetables, nuts and vegetable oils that are high in unsaturated fats (Table 1).^15^^,^^16^

**Table 1:** Summary of evidence and practice across key policy considerations for fiscal interventions to improve diets and health, 2017

Topic	Policy consideration	Current evidence base	In practice^a^	Research opportunities
Identifying targets for fiscal policy	Targets relevant to prevention of NCDs	Strong epidemiological evidence for increased risk of NCDs associated with consumption of added sugars, red and processed meats refined grains, salt, sugar-sweetened beverages and trans fat and of decreased risk of NCDs associated with consumption of fish, fruits, legumes, minimally-processed whole grains, non-starchy vegetables, nuts and vegetable oils that have high concentrations of unsaturated fats.	By 2017, 25 jurisdictions had implemented taxes on non-core foods – mainly SSBs but also snack/packaged foods high in salt, sugar and/or fat. There has been some implementation of targeted subsidies on healthy foods such as fruit and vegetables.	In-practice assessment of response of consumers, in terms of substitution.
	Effectiveness in improving diets	SSBs associated with the highest price elasticity estimates. Nutrient-based taxes most effective when broad-based – e.g. when targeted at energy-dense, nutrient-poor foods rather than a single nutrient. Fruit and vegetable subsidies increase consumption. So-called healthy food subsidies may increase consumption but may possibly also increase overall caloric intake.	Emerging evidence from detailed evaluations of taxes on SSBs in Mexico and Chile showing significant reductions in consumption of the beverages and an increase in consumption of drinking water. A public-health tax in Hungary, on foods with high salt, fat and/or sugar concentrations, reduced consumption of processed food by 3.4%. Evidence from South Africa of increased daily consumption of fruit and vegetables – by 0.38 and 0.64 of a serving per person with subsidies of 10% and 25%, respectively.^12^	In-practice studies of impact of fiscal policy intervention on consumption, energy intake, body weight and disease outcomes.
	Administrative elements	Targets of fiscal policy must be feasible to identify within the existing taxation system.	Implemented taxes have focused on clearly defined foods or beverages – mostly on SSBs. In Hungary, a tax was based on nutrient profiling of processed, packaged foods.	Context-specific research to identify feasible approaches to identifying foods or beverages for taxation, within existing tax systems.
Policy design to maximize impact	Setting the tax rate	Rationale for taxation is internalizing the external costs associated with the foods and beverages linked to increased risk of NCDs. Taxation rate should be commensurate with this. Evidence indicates that taxes of at least 20% are most effective in changing consumption. If taxes are graduated according to the concentration of an unhealthy nutrient in a processed food, the reformulation of that food is made more likely.	Implemented taxes tend to be around 10% by value, in some cases because higher levels provoke too much political opposition. Much higher taxes – of 100% on energy drinks and 50% on carbonated drinks – have been implemented in Saudi Arabia and the United Arab Emirates. In the United Kingdom, the upcoming levy on SSBs will be graduated, based on sugar content. Implemented subsidies vary widely, depending on target group, and budgetary limitations appear to be the main associated issue.	In-practice assessment of the impact of different tax rates on consumption and reformulation.
	Selecting the taxation mechanism/policy tool	Global recommendations for tax reform favour use of excise taxation. A targeted excise or production tax – set according to volume or weight rather than price – reduces incentives for consumers to substitute towards cheaper brands.	Excise taxes, sales taxes and taxes on commercial production are the main approaches to operationalizing taxation to improve diets. Targeted subsidies have been embedded in social welfare programmes, targeted at remote populations or provided by private health insurance programmes. In some countries, implicit subsidies have been provided through removal of import tariffs on fruit and vegetables.	Relative efficiency of different taxes. Impact on supply chains.
	Geographical scope	Industry data indicate that tax avoidance can occur through consumers purchasing untaxed foods or beverages beyond the jurisdiction where the taxation is applied.	Cross-border purchasing to avoid taxes has been more commonly seen when taxation has been implemented over a relatively smaller area.^13^	Impact and scale of cross-border purchasing.
	Combination with other interventions	Effects of fiscal policy may be enhanced by complementary interventions, such as education. Other policies may support or undermine diet-based fiscal policies.	A Mexican tax on SSBs was accompanied by consumer awareness campaign, to raise public support for the intervention and support further behavioural change. Agricultural subsidies in the USA, on corn and sugar, have been posited to increase obesity.	Context-specific research to identify impacts of complementary or contrary interventions.
Fiscal considerations	Impact on equity and regressive taxes	Potential to minimize regressive taxes and impacts on equity, through using revenue for health and social purposes, applying taxes to non-core foods and combining taxes with subsidies.	Revenues from diet-related taxation have been earmarked for nutrition programmes, a health promotion foundation, pre-school education, provision of clean drinking water and a public health campaign. Evaluation of the soda tax in Berkeley found that consumption decreased substantially in neighbourhoods of low socioeconomic status.	Effect of hypothecation on health and social outcomes. Combined effect of taxes and subsidies.
	Revenue	Given price elasticity estimates, taxes are likely both to reduce consumption and raise revenue.	Substantial revenue has been raised from taxes implemented in several jurisdictions – e.g. French Polynesia, Mexico and Nauru. Estimates of potential revenue encouraged political support for diet-related fiscal policies in French Polynesia, Mexico and Samoa.^10^	Estimates of impact of taxation on revenue.
	Cost–effectiveness	Diet-related taxes and subsidies are likely to be highly cost–effective.	There has been little cost–effectiveness analysis of implemented policies.	In-practice evaluation of impact and cost–effectiveness.
Employment and industry	Impact on employment and welfare	Potential negative impacts of fiscal policy on employment – and thus population well-being – via the reduced consumption of highly profitable products.	In South Africa, industry lobbied strongly against fiscal policy on the basis of job losses but independent review found these losses were overestimated.	In-practice evaluation of impact on employment.
	Response and role of industry	Industry likely to mediate the impact of the tax through strategic pricing – but little information on extent and impact of this.	In Berkeley, 43% of the tax on SSBs was passed onto consumers in the form of higher retail prices.^14^	Better understanding of detailed impacts of fiscal policies on price – and the role of industry in mediating this effect.

NCDs: noncommunicable diseases; SSBs: sugar-sweetened beverages.

^a^ Unless indicated otherwise, the information in this column comes from the World Cancer Research Fund’s NOURISHING framework.^6^

Overall, the evidence indicates that, if we are to reduce the risks of diet-related noncommunicable diseases, we would be better altering overall diet rather than focusing on the consumption of individual food items.^16^ Thus, the relative healthfulness of any nutrient needs to be judged in the context of the entire diet. Policy-makers also need to differentiate between so-called core foods, the consumption of which is recommended by government dieticians, and non-core or discretionary foods that are generally considered to be less beneficial. For example, although unsweetened milk and a soft drink may contain a similar number of calories per litre, only the milk may be considered a core food because of its calcium content. Most tax-based policies to improve diets have focused on non-core foods or beverages, particularly sugar-sweetened beverages (Table 1).

#### Improving diets

The evidence indicating that diet-related fiscal policies can benefit public health is focused on sugar-sweetened beverages, which are one of the more price-elastic targets of taxation that have been examined. Estimations show that such beverages have a mean price elasticity of about −1, indicating that a 1% increase in the retail price of such beverages should lead to a reduction in consumption of about 1%.^17^^,^^18^ In practice, a tax of about 10% on sugar-sweetened beverages in Mexico and an increase in the tax on such beverages in Chile, from 13% to 18%, are both estimated to have reduced national consumption by about 7%.^19^^,^^20^

Although taxes targeting a single nutrient can reduce consumption of that nutrient, they can also lead to increases in the consumption of other, less healthy nutrients and to decreases in fruit and vegetable intake.^21^ In contrast, broader taxes on energy-dense, nutrient-poor foods are harder to evade and may have a greater and more consistent beneficial impact on the consumption of such foods and body weight.^5^^,^^18^^,^^21^ The overall price elasticity of such diverse foods is difficult to estimate. A meta-analysis indicated that each 10% increase in the retail price of so-called fast foods and other unhealthy foods led to reductions in consumption of 3% to 9%.^5^

Subsidies on fruit and vegetables have been found effective in increasing consumption.^18^^,^^21^^,^^22^ Although broader subsidies on healthy foods have also been successful in increasing consumption of the target foods, they have also been associated with an overall increase in food intake and thus caloric intake.^21^^–^^23^ In South Africa, 10% and 25% subsidies on fruit and vegetables led to mean increases in daily fruit and vegetable intakes of 0.38 and 0.64 of a serving per person, respectively.^12^

A combination of taxes on unhealthy foods and subsidies on healthy foods may be effective in changing consumption in the desired direction and also reduce potential unintended consequences – e.g. increases in the consumption of non-targeted foods.^18^^,^^21^

While the evidence for the beneficial impact of diet-related fiscal policies on consumption and diets has mounted, the evidence for the effects of such policies on total energy intake, body weight and disease outcomes remains limited and inconclusive.^17^^,^^18^ The evaluation of the full benefits of such policies can be complicated by food substitution and the cost and logistical problems of long-term follow-up. However, a recent mathematical model based on the relevant data from South Africa indicated that diet-related fiscal policies could have substantial health benefits in the long term.^24^

#### Administrative considerations

Appropriate targets for diet-related fiscal policies must be identifiable within existing taxation systems.^11^ In consequence, policies targeting clearly defined foods, e.g. soft drinks, may be easier to implement than more complex policies that target multiple nutrients across a range of foods, particularly in low-resource settings. To maximize the impact of a simple tax, maintaining a wide tax base that includes most products containing the target nutrient is important. In most countries, for example, a tax on sugar-sweetened beverages would be a tax on a large proportion of discretionary sugar intake. However, the fact that Denmark managed to implement a tax based on the percentage of saturated fat in foods indicates that more complex, nutrient-focused taxes may also be administratively feasible in some settings.^7^

### Maximizing impact

#### Setting tax/subsidy rates

One motivation for taxing energy-dense, nutrient-poor foods is to internalize the external costs that such foods impose through the health system and/or through lost productivity. As much of the costs of noncommunicable diseases associated with a poor diet are borne by society and lower social welfare, there is an economic rationale for taxing such foods.^13^ The economic perspective is that the amount of the tax should be equal to the marginal external costs, e.g. those associated with additional medical care and higher job absenteeism, that would otherwise be imposed on society. The marginal external costs associated with specific energy-dense, nutrient-poor foods still need to be estimated. The public health perspective may be that taxes should be set sufficiently high to cause a meaningful reduction in consumption, even if that tax exceeds the external costs. Estimations show that a tax of at least 20% and/or a subsidy of at least 10% can generate meaningful changes in consumption.^2^ In practice, most of the implemented taxes are around 10%, although Saudi Arabia and the United Arab Emirates have recently implemented taxes of up to 100% on sugar-sweetened beverages (Table 1).^6^

Ideally, the level of the diet-related taxation of a food product should increase with increasing content of target nutrients in a product. This would give consumers an incentive to switch to healthier products and give producers of processed foods an incentive to improve the healthiness of their products. The scheduled levy on beverages in the United Kingdom of Great Britain and Northern Ireland will be graduated according to the added-sugar content of the beverage. The details of the tax were announced in advance, so that manufacturers could start reformulating their products in anticipation of the tax.^25^

#### Mechanism of taxation

Excise taxes, sales taxes and taxes on commercial production are the main options for taxation to improve diets (Table 1).^23^^,^^26^ The benefit of targeted excise or production taxes is that such taxes are applied by volume of liquid or weight of food. In contrast, taxes as a percent of price create incentives for consumers to substitute toward cheaper products instead of healthier products.. Excise or production taxes are also preferable because, compared with a sales tax, they are more likely to be built into the shelf price that consumers see when making their purchase.^26^ This is useful because, all else being equal, the more visible the tax, the greater the behavioural change made in response to it.^27^ Global recommendations for tax reform also favour the use of excise taxation for commodities, to complement broad-based value-added taxes.^11^

There is less agreement on the best mechanisms for health-related subsidies. In practice, subsidies have been embedded in social welfare programmes, targeted at remote populations or provided by private health insurance programmes (Table 1).

#### Geographical scope

Although most of the diet-related fiscal policies that have been implemented are national, some individual municipalities have enacted their own systems of taxation to improve diets. Although policies on such a small geographical scale can be tailored to particular communities and circumstances, the corresponding taxation is easier to evade through cross-border shopping (Table 1).^13^^,^^14^ Such shopping may also be a problem in small countries with open borders, such as Denmark.^7^

#### Combination with other interventions

The effectiveness of fiscal interventions may be enhanced through efforts to educate consumers and improve public awareness that the target has either been taxed, because it is an unhealthy product or subsidized, because it is a healthy product.^5^^,^^21^^,^^22^^,^^28^^,^^29^ This is in line with recommendations from WHO that emphasize the benefits of implementing a comprehensive package of nutrition interventions to improve diets and health.^1^ In Mexico, implementation of a tax on sugar-sweetened beverages was accompanied by consumer awareness campaigns that were designed to support behavioural change and a structural intervention to increase the availability of potable water.^30^

Existing policies in other sectors might impact the price of food and thus work in combination, or unintentionally undermine, diet-based fiscal policies. For example, agricultural policies and food aid programmes can affect the relative prices of food.^31^ In addition, many low- and middle-income countries implement price-control policies that limit the consumer prices of a basket of essential goods, which can include unhealthy commodities, such as fat, salt and sugar.

### Fiscal considerations

#### Equity and regressive tax

The potential impact of fiscal policies on equity is a concern.^11^ Policy-makers must consider whether a proposed fiscal policy will result in any restriction of freedoms and/or exacerbate inequalities by disproportionately affecting some groups of individuals.^32^^–^^34^ Taxes on food and beverages are likely to be regressive because, compared with their richer counterparts, people on low income spend higher percentages of their incomes on such products.^21^^,^^35^ From an ethics perspective, these impacts on inequalities need to be balanced against the effectiveness of the intervention and whether the population group most affected by taxes on consumption receives any reciprocal benefit for their tax burden.

Studies on the ethical implications of using taxation as a public health tool, at least in the context of tobacco control, indicate that regressive taxes and the restriction of freedoms are probably justified by the potential health benefits, ^36^^,^^37^ especially if the revenue from the taxes is used to fund support services that assist people to stop smoking. In relation to the restriction of freedoms, it is important to be clear that such taxes do not represent a complete prohibition of choice or denial of freedom. However, by increasing the retail prices of tobacco and tobacco products, they do restrict or limit the capacity for an individual with finite economic resources to choose in accordance with their desire.

It has been proposed that the revenues from food taxes be used to enhance or amplify the health benefits of the taxation, reduce potential inequalities and improve public acceptability of the taxation.^21^^,^^35^ In the United States revenues from soft-drink tax in the Californian city of Berkeley are earmarked for nutrition programmes for schoolchildren and other health-related programmes sponsored by community groups.^6^

Low-income consumers may experience disproportionate health benefits through larger reductions in consumption, particularly when there is health-related taxation on soft drinks.^4^^,^^17^^,^^18^^,^^21^ In Berkeley, substantial decreases in the consumption of sugar-sweetened beverages were found among consumers with low socioeconomic status.^38^ If taxes are applied to non-essential foods rather than core foods and combined with subsidies on core foods, consumers would have much scope to change their consumption, by substitution, and impacts from regressive taxes could be minimized.

#### Revenue

Revenue is a critical topic for policy-makers considering the implementation of new taxes. Recently, the positive impact of Mexico’s soft-drink tax on revenue, as well as the potential to allocate revenue to improving the supply of safe drinking water, has proved a key factor in the government’s decision to maintain the tax in the face of continued industrial opposition.^39^

#### Cost–effectiveness

Taxation to improve diets is likely to be very cost–effective.^40^ In Brazil, China, India, Mexico, the Russian Federation, South Africa and United Kingdom (England), a study has investigated methods to tackle unhealthy diets, physical inactivity and obesity. The results indicated that, in terms of cumulative effectiveness and cost saving, fiscal measures that reduced the prices of fruit and vegetables or increased the prices of foods high in fat were always cost–effective.^41^

Evidence on the cost–effectiveness of subsidies, which are often perceived as a drain on government budgets, is scarce. However, a simulation based on mathematical models indicated that, in the United Kingdom, a 10% subsidy on healthy foods would be cost–effective, because of the cost savings in government-provided health care.^42^

### Concerns of employment and industry

#### Impact on employment and welfare

The beverage, food and sugar industries have actively lobbied against diet-related taxation on foods and beverages.^7^^,^^43^ Although they have claimed that such taxation will lead to job losses, some of their estimates of the potential negative impacts on employment appear too high. In South Africa, for example, concerns about employment were the primary arguments against a tax on sugar-sweetened beverages. However, independent estimates of the potential effects of such a tax on employment, which considered development of alternate markets, were substantially lower than those quoted by industry actors.^9^ A recent analysis in the United States indicated that a sugar-sweetened beverage tax could actually increase employment overall, as consumers reallocate their spending and the government uses revenue from the taxation to generate employment.^44^ Subsidies designed to improve diets and health – particularly those applied to agricultural goods – are likely to be even more directly positive in their impact on employment.

#### Industry’s response and role

Various firms, including food manufacturers, distributors and retailers, are likely to play a critical role in determining the effects of food taxes. For example, each may absorb some of the tax rather than pass it on to consumers in the form of higher prices. Although there is limited relevant evidence available, the role of industry in mediating the effect of taxation on consumers is likely to be substantial.^21^^,^^22^ In Berkeley, only 43% of the soda tax that was levied on distributors was passed on to consumers in the form of higher retail prices.^14^ However, early evidence from Philadelphia, United States, indicates that a much higher percentage of the tax was passed on to consumers in that city.^45^

Food industry actors are primarily concerned about the effect of food taxation on consumer demand, sales and profits.^46^ Consideration of market dynamics and the availability of close substitutes underpin strategic pricing.^47^ Pricing decisions are also affected by internal factors such as costs, marketing objectives, the marketing mix strategy and other organizational considerations.^48^ As a result, taxation of products for which there are close, untaxed substitutes offers greater opportunity for industry to use advertising or discounts to encourage customers to switch to the untaxed healthier substitutes.

Food manufacturers can play a key role in reformulating their products to reduce or even eliminate diet-related taxation on them. Well designed taxes or subsidies, which vary based on the content of unhealthy nutrients in foods, give strong incentives for such reformulation. Reformulation that increases the healthiness of foods has already occurred in response to more detailed labelling on packaged foods and the addition of calorie numbers to menus.^49^

## Conclusions

Well designed taxes and subsidies can change the prices, purchase and consumption of target foods, although the effects on overall diet and health are less clear. To maximize impact, the ideal tax needs to be implemented on a large geographical scale, to be designed with graduated thresholds for the nutrients of concern and should cover a broad range of non-core food items that are energy-dense and nutrient-poor. The most effective structure of such a tax is likely to be an excise tax that is applied on the basis of volume or weight and included in shelf prices.

Factors relevant to political economy underlie many of the policy considerations identified in this review. An understanding of the political and corporate environment in which the adoption, or blocking, of diet-related fiscal policies occurs can enable identification of the conditions under which governments may be more likely to make such policies a political priority.^50^ Further research into the political economy of fiscal policies to improve diets and health, and research into the impact of diet-related taxation on industry and the role of industry in mediating the impact on consumers, would also support the design of more effective fiscal policies.
